# N6-Methyladenosine Modification Patterns and Tumor Microenvironment Immune Characteristics Associated With Clinical Prognosis Analysis in Stomach Adenocarcinoma

**DOI:** 10.3389/fcell.2022.913307

**Published:** 2022-06-15

**Authors:** Zhang Meijing, Luo Tianhang, Yang Biao

**Affiliations:** ^1^ Department of Oncology, Changhai Hospital, Second Military Medical University, Shanghai, China; ^2^ Department of General Surgery, Changhai Hospital, Second Military Medical University, Shanghai, China

**Keywords:** stomach adenocarcinoma, N6-methyladenosine, tumor microenvironment, immunotherapy, mutation burden, microsatellites instability

## Abstract

**Background:** N6-methyladenosine (m6A) modification is a part of epigenetic research that has gained increasing attention in recent years. m6A modification is widely involved in many biological behaviors of intracellular RNA by regulating mRNA, thus affecting disease progression and tumor occurrence. However, the effects of m6A modification on immune cell infiltration of the tumor microenvironment (TME) are uncertain in stomach adenocarcinoma (STAD).

**Methods:** The Cancer Genome Map (TCGA) database was used to download transcriptome data, clinicopathological data, and survival data for m6A-regulated genes in 433 STAD tissues that meet the requirements of this study. GSE84437 data were obtained from the Gene Expression Omnibus (GEO) database. The correlation between 23 m6A regulated genes was analyzed using R software. Sample clustering analysis was carried out on the genes of the m6A regulatory factor, and survival analysis and differentiation comparison were made for patients in clustering grouping. Then, the Gene Set Enrichment Analysis (GSEA), the single-sample GSEA (ssGSEA), and other methods were conducted to assess the correlation among m6A modification patterns, TME cell infiltration characteristics, and immune infiltration markers. The m6A modification pattern of individual tumors was quantitatively evaluated using the m6A score scheme of the principal component analysis (PCA).

**Results:** From the TCGA database, 94/433 (21.71%) samples were somatic cell mutations, and ZC3H13 mutations are the most common. Based on the consensus, matrix k-3 is an optimal clustering stability value to identify three different clusters. Three types of m6A methylation modification patterns were significantly different in immune infiltration. Thus, 1028 differentially expressed genes (DEGs) were identified. The survival analysis of the m6A score found that patients in the high m6A score group had a better prognosis than those in the low m6A score group. Further analysis of the survival curve combining tumor mutation burden (TMB) and m6A scores revealed that patients had a significantly lower prognosis in the low tumor mutant group and the low m6A score group (*p* = 0.003). The results showed that PD-L1 was significantly higher in the high m6A score group than in the low score group (*p* < 2.22e-16). The high-frequency microsatellite instability (MSI-H) subtype score was significantly different from the other two groups.

**Conclusions:** This study systematically evaluated the modification patterns of 23 m6A regulatory factors in STAD. The m6A modification pattern may be a critical factor leading to inhibitory changes and heterogeneity in TME. This elucidated the TME infiltration characteristics in patients with STAD through the evaluation of the m6A modification pattern.

## Key Points

• This study systematically evaluated the modification patterns of 23 m6A regulatory factors in STAD.
• This study revealed that m6A modification is significantly associated with TME diversity and complexity.
• The m6A score has the potential in predicting the clinical response of PD-L1 blockade.
• Quantitative evaluation of the m6A modification patterns of individual tumors will strengthen our understanding of TME characteristics and promote effective immunotherapy strategies.

## Introduction

Traditional epigenetics research focuses on DNA methylation, histone modification, non-coding RNA, and chromatin remodeling (Boccaletto et al., 2018). Methylation of N^6^ adenosine (m6A) is the primary methylation in eukaryotic mRNA and long non-coding RNA and is regulated by methyltransferases (writers), demethylases (erasers), and binding proteins (readers) (Roundtree et al., 2017; Shen et al., 2022). Substantial m6A methylation is detected in the “RRACH” base sequence through high-throughput sequencing and bioinformatics analysis (Meyer et al., 2012; Jenjaroenpun et al., 2021). It is also rich in areas, such as stop codon and 3′-untranslated region (UTR) (Dominissini et al., 2012). m6A methylation regulates the translation of mRNA, nuclear transport, and degradation, thereby determining the entire life process of mRNA. Other RNAs in the cell, including transport RNA (tRNA), ribosome RNA (rRNA), and long non-coding RNA (lncRNA), also have a large amount of m6A methylation. Moreover, studies have shown that m6A methylation is involved in the complex and delicate regulation of critical functional genes, especially in the development of tumors. Therefore, studying the role of m6A modification is crucial to clarify the tumor mechanism and clinical treatment.

In recent years, immunotherapy usage has revolutionized the regulation of the immune system to exert the anti-tumor effect for the treatment of malignant tumors. However, immunotherapy offers lasting survival benefits in only 20%–30% of patients in clinical practice (Tabernero et al., 2018). Most patients face immunotherapy resistance. Therefore, the major issue of immunotherapy is the lack of accurate prediction of the dominant population and the systematic research and response of drug resistance mechanisms, resulting in excessive or insufficient immunotherapy. Recent studies on the interaction between tumors and tumor microenvironments (TMEs) have provided novel opportunities for immunotherapy. Tumor cells induce immune escape by inhibiting the response and function of infiltrative immune cells by suppressing signaling pathways, such as the programmed cell death protein 1/programmed cell death protein-ligand 1 (PD-1/PD-L1) (Chen and Flies, 2013; Beatty and Gladney, 2015; Xiong et al., 2022). In addition, the metabolic reconstruction of tumor cells consumes excess sugar, and amino acids competitively deprive T cells of the required nutrients, promoting the deactivation and immunosuppression of T cells (Li and Zhang, 2016). On the other hand, the recruitment and amplification of immunosuppressive cells in TME, such as T-regulatory lymphocytes (Tregs), tumor-associated macrophages (TAMs), and myeloid-derived suppressor cell (MDSCs) is also one of the primary mechanisms that induce immunosuppressive TME (Davis et al., 2016; Yang et al., 2021). The immune status of TME is a critical factor affecting tumor progression. TME presents a differentiated degree of immune activation under the action of specific immune cells or molecules. The targeted TME immunotherapy involves the intervention of non-tumor cells and components and can transform the immune response from tumor promotion to tumor suppression (Zou, 2018). Similarly, the combination of anti-tumor and multi-target immunotherapy drugs can avoid adaptive resistance and improve tumor prognosis and survival significantly (Lambrechts et al., 2018; Lee et al., 2020).

Stomach adenocarcinoma (STAD) is one of the most common malignant tumors worldwide. Statistically, the morbidity of malignant tumors ranks fourth, and the mortality rate is third (Chen et al., 2016). STAD is a multi-step, multi-factor disease similar to other malignant tumors. Some studies demonstrated that m6A is closely related to the immune status of TME. The interaction among various mechanisms formed a complex network that promoted the development of tumors (Liu et al., 2022). Reportedly, METTL3 is high in STAD patients and increases with the progression of tumor stages and grades (Wang et al., 2020; Yang et al., 2020). Strikingly, METTL3 knockout reduces the expression of EMT-related proteins, thereby inhibiting STAD cell proliferation and migration. Some studies have reported that high expression of METTL3 is significantly associated with the clinicopathological characteristics and poor survival in STAD patients and that knocking out METTL3 can inhibit cell proliferation, migration, and invasion. Further RNA-seq and m6A-seq analysis showed that METTL3 promotes STAD development via m6A modification and regulates key proteins on MYC target genes such as *MCM5* and *MCM6*. In addition, it has also been shown that EIF3B promotes the migration and invasion of tumor cells by regulating EMT and STAT3 signaling pathways (Wang et al., 2019). However, the tumor immunosuppressive microenvironment is a complex network regulated by multiple immunosuppressive signals that are constantly changing dynamically; hence, targeting a single immunosuppressive signal alone does not achieve long-term efficacy. Therefore, a multi-targeted immunotherapy strategy is essential to understand the m6A modification combined with TME to screen immunotherapy-sensitive markers. Also, exploring new immunotherapy targets in the future is imperative.

In this study, we analyzed the differences in the expression of m6A using STAD sample transcriptome data and related mutation data from The Cancer Genome Atlas (TCGA) and Gene Expression Omnibus (GEO) databases. Then, we evaluated the correlation between m6A modification patterns and TME cell-infiltrating characteristics. The TME characteristics of the three m6A modification modes and the three immune phenotypes were consistent. We also established a scoring system for m6A modification patterns to quantify the patients individually. The correlation between survival time, clinical response to immunotherapy, tumor mutation burden (TMB), and microsatellite instability (MSI) was assessed based on the quantification of m6A modification patterns. These results confirmed that m6A methylation plays a critical role in the development of STAD through TME, creating opportunities to predict the prognosis of clinical STAD patients and explore new targeted treatments.

## Methods

### Data Collection

TCGA and GEO downloaded RNA-seq transcription group and clinical data from 433 STAD patients. This sample selected GSE84437 cohorts with complete clinical information and follow-up data for final inclusion in the study (Yoon et al., 2020). The Fragments Per Kilobase of exon model per Million mapped fragments (FPKM) format data were downloaded using the “fpkm” function in R to convert them to the transcripts per kilobase million (TPM) for the next analysis (Zhao et al., 2020). Then, we performed copy number variation (CNV) data analysis of qualified TCGA-STAD data. The CNV data were downloaded from the UCSC Xena database (http://xena.ucsc.edu/). Data analysis and collation were carried out using R software (version 3.6.1).

### m6A RNA Methylation Regulator for Stomach Adenocarcinoma in The Cancer Genome Map

We retrieved the literature related to m6A methylation modification, and a total of 23 acknowledged m6A regulator genes were curated and analyzed to identify distinct m6A methylation modification patterns (Chen et al., 2019; Zaccara et al., 2019; Liu et al., 2022; Mobet et al., 2022). Based on the mRNA expression data available in TCGA, we analyzed 23 m6A RNA methylation regulators (METTL3, METTL14, METTL16, WTAP, VIRMA, ZC3H13, RBM15) RBM15B, FTO, ALKBH5, YTHDC1, YTHDC2, YTHDF1, YTHDF2, YTHDF3, HNRNPC, FMR1, LRPPRC, IGF2BP1, IGF2BP2, IGF2BP3, HNRNPA2B1, and RBMX) data. The expression and the correlation of mRNA of 23 m6A methylation regulators were analyzed. The correlation between PD-1, PD-L1, and m6A RNA methylation regulatory factors was established, and the corresponding heat maps were drawn. ConsensusClustPlus package was used for consistency clustering. The optimal clustering (k-value) was evaluated based on the clustering score of the cumulative distribution function (CDF) curve. The R software ConsensuClusterPlus package was used to draw a heat map and verify the differences using Wilcox rank test through limma packets.

### Unsupervised Consensus Clustering of 23 m6A Regulators and Functional Analysis

The m6A differential genes were analyzed using GSEA. *p* < 0.05 and the false discovery rate (FDR) < 0.05 indicated a significantly enriched gene set. GSVA package was used for differential analysis of m6A modification pattern activity (Foroutan et al., 2018). ConsensusClustPlus package was utilized for consistency clustering. We also used the clusterProfiler package for Gene Ontology (GO) and Kyoto Encyclopedia of Genes and Genomes (KEGG) pathway enrichment analyses.

### Immune Infiltrating Cell Analysis

TME immune infiltration cells used CIBERSORT (a bioinformatics algorithm) to assess the correlation between m6A expression and the abundance of tumor-infiltrating immune cells (TIIC), including CD4-T cells, CD8-T cells, and macrophages. Immune infiltration cells in TME for STAD were analyzed using ssGSEA (Hanzelmann et al., 2013). The differences between the m6A modification patterns and immune cells were analyzed using differential analysis of immune cells.

### Differentially Expressed Genes Among the m6A Phenotypes

We used the R limma package to screen for m6A Differentially Expressed Genes (DEGs) between different m6A phenotypes. The adjusted *p*-value < 0.05 was set The significance filtering criteria of DEG. GO and KEGG analyses of the differential genes were carried out using the R clusterProfiler and enrichplot packages. The results are shown in bar and bubble charts.

### Prognostic Signature of m6A-Related Genes

The differences in genes in different m6A clusters in STAD patients were quantified using the principal component analysis (PCA). First, we extracted the overlapping genes from DEGs. Then, consensus clustering algorithms were employed to test the number and stability of gene clusters. Finally, PCA was used to analyze the different genes related to prognosis. Next, we established m6A-related features based on the results of the analysis. In addition, PCA maximized the integrity of the data. This method was used to assess the m6A gene characteristics of STAD patients, termed the m6A score. The m6A score was calculated using the following formula (Sotiriou et al., 2006; Zeng et al., 2019): m6Ascore = ∑(PC1_i_ + PC2 _i_), where i represents the expression of m6A-related genes. Patients were divided into high- and low-score groups based on the ranking statistics. Subsequently, we used immunophenoscore (IPS) to detect the characteristics of the tumor immune landscape (Charoentong et al., 2017). IPS was used to detect the efficacy of anti-CTLA-4 and anti-PD-1 treatment regimens and calculated using four types of immune-related genes: MHC molecules (MHC), immunomodulators (CP), and effector cells (EC), and suppressor cells (SC).

### Statistical Analysis

Statistical analysis was carried out using R 3.6.1 software. The comparison of the expression levels of core genes between different genotype groups did not show normal distribution, as shown in the median and quartile number of spacings (P25, P75). Wilcoxon testing was used for group comparisons. A single-factor Cox analysis of 23 m6A methylation regulators used the survival package and screening condition *p* < 0.05 to determine the correlation between RNA m6A methylation regulation in STAD tumor tissue and prognosis related to mRNA expression. The log-rank Kaplan–Meier survival curve analyzed the progenitive correlation. Spearman’s test was used in the analysis of the correlation between the core genes and the infiltration degree of different immune cells. 0.1 ≤ |r| ≤ 1.0 was defined as relevant. *p* < 0.05 indicated statistically significant differences.

## Results

### Genetic Variation Profile of m6A Regulators in Stomach Adenocarcinoma

**FIGURE 1:**
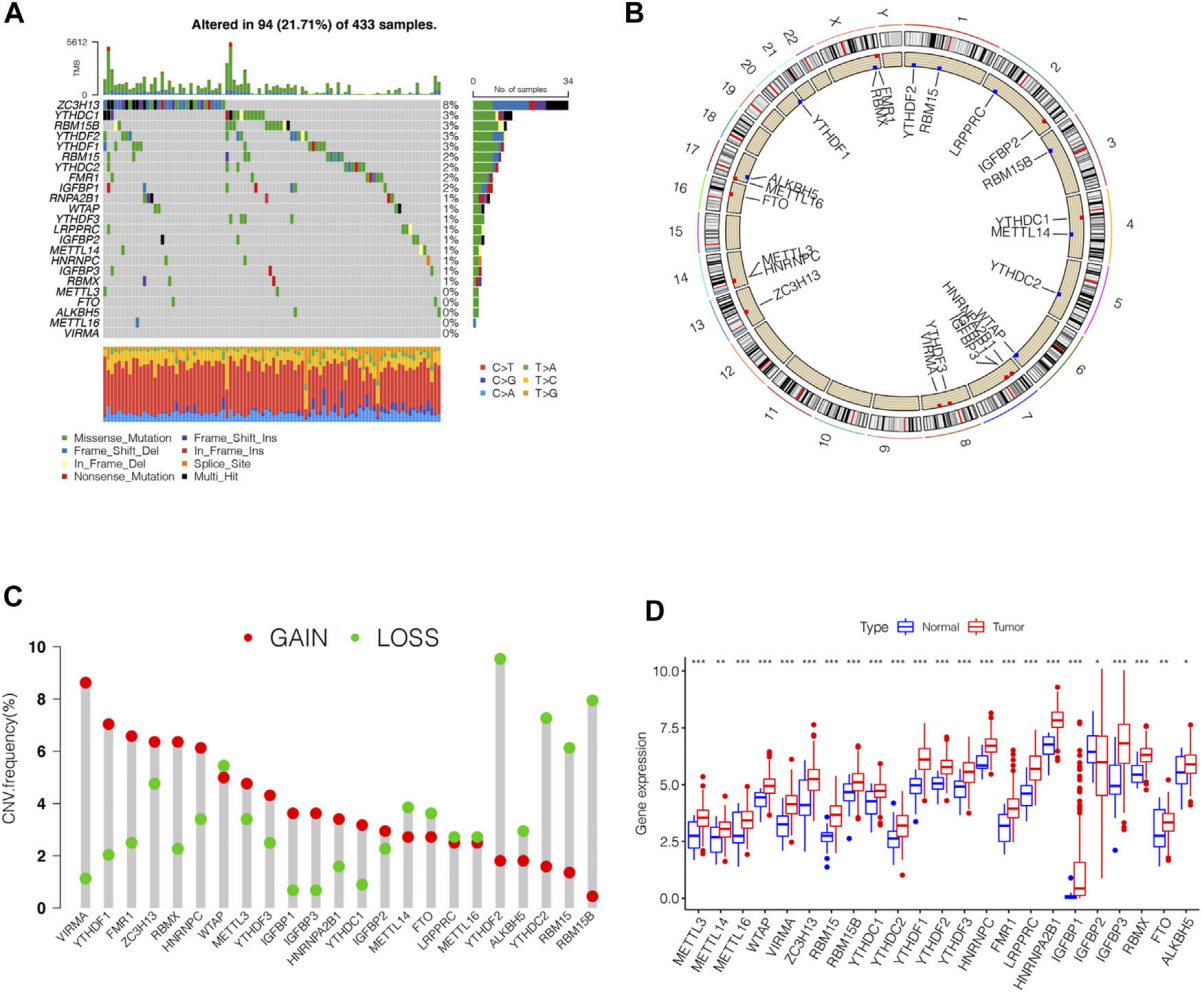
Genetic variation profile of m6A regulators in STAD. **(A)** Mutation frequency of the m6A regulators of stomach adenocarcinoma patients in the TCGA-STAD cohort. **(B)** Location of CNV changes of 23 m6A regulators on the chromosome. **(C)** A histogram plotting the CNV mutation frequency of each gene obtained by statistical analysis of the copy number of m6A. The abscissa was the m6A-related gene, and the ordinate was the mutation frequency. **(D)** The box plot of m6A differential expression analysis in the tumor and normal samples. The asterisks represented the statistical *p* value (^∗∗∗^ *p* < 0.0001, ^∗∗^ *p* < 0.01, ^∗^ *p* < 0.05).

In this study, we identified the role of 23 m6A regulation genes in STAD, including 13 readers, 8 writers, and 2 erasers. After downloading the relevant mutation data from the TCGA database, we identified somatic cell mutations in 94/433 (21.71%) samples; among these, ZC3H13 mutations were the most common (Figure 1A). The position of the m6A regulator CNV mutation on the chromosome is observed on the Circos plot (Figure 1B). Further analysis of CNV mutations revealed that VIRMA, YTHDF1, and FMR1 showed extensive CNV amplification, while YTHDF2, RBM15B, and YTHDC2 showed widespread CNV deficiency (Figure 1C). Next, we investigated whether changes in CNV altered the expression of regulatory m6A in STAD and found that almost all m6A regulators were expressed significantly higher in STAD tissue than in normal tissue, except IGFBP2 (Figure 1D). These results indicated significant genetic variation characteristics of m6A regulatory in STAD, suggesting that the complexity of m6A modification and tumor heterogeneity play a fundamental role in STAD development.

### Identification of m6A Methylation Modification Patterns in Stomach Adenocarcinoma

**FIGURE 2:**
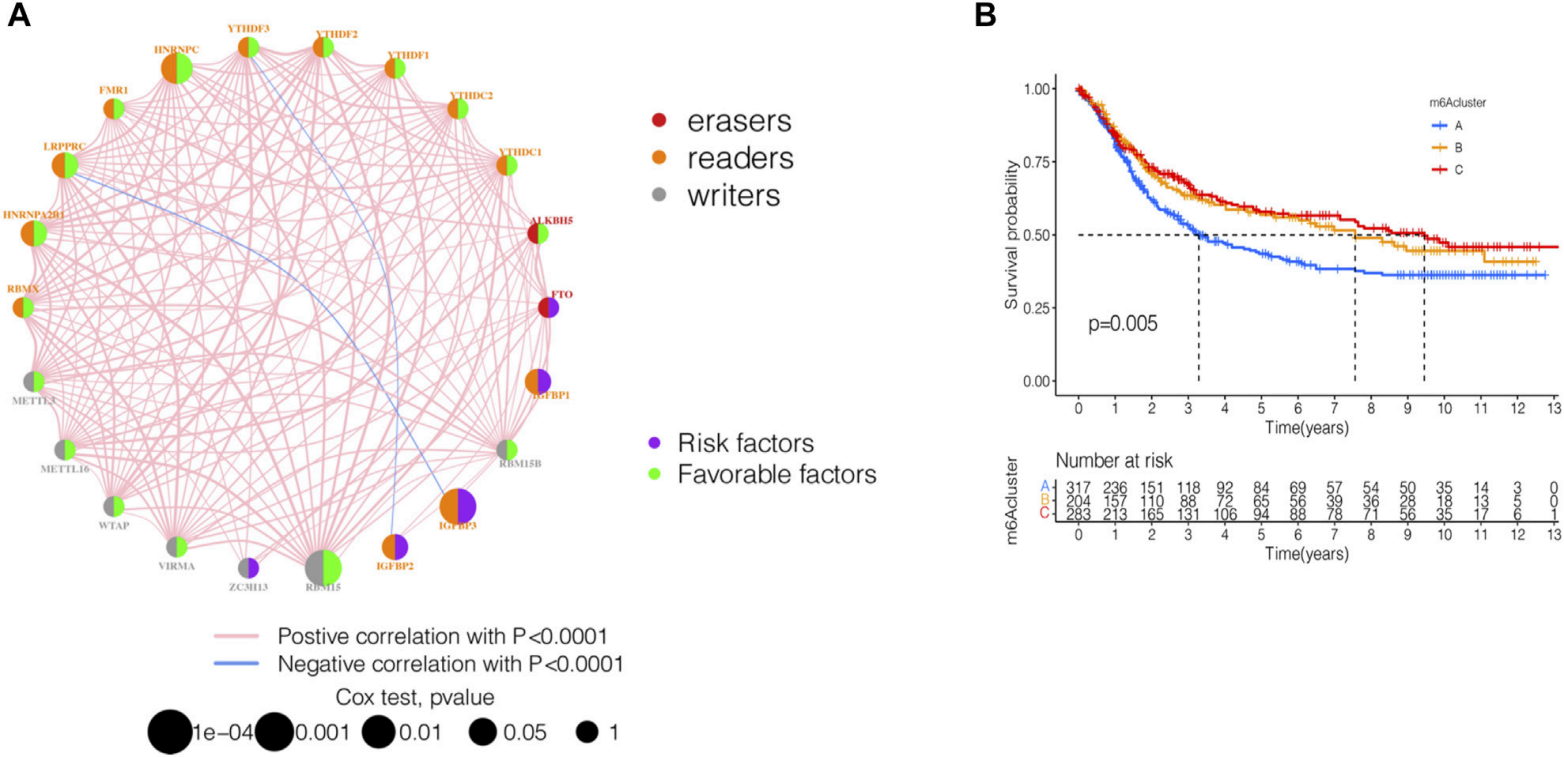
Identification of m6A methylation modification patterns in STAD. **(A)** Interaction of 23 m6A regulators and their prognostic significance in STAD. The circle size represented the effect of each regulator on the prognosis, and the range of values calculated by Cox test was *p* < 0.001, *p* < 0.01, *p* < 0.05, and *p* < 1, respectively. **(B)** Kaplan–Meier curves of m6A modification patterns. Kaplan-Meier curves with Log-rank *p*-value 0.005 showed a significant survival difference among three m6A modification patterns.

GSE84437 dataset with complete survival and clinical information in the GEO database and the TCGA-STAD (*n* = 443) data were merged for the analysis of the correlation of tumor mutation load. The results showed that TTN and TP53 had the highest TMB. The top 10 genes of the mutant burden are shown in Supplementary Table S1. According to the most common mutation of ZC3H13, the m6A regulator genes were divided into ZC3H13 wild-type and ZC3H13 mutation type. The results of the TMB and expression correlation analysis of the m6A regulator genes are shown in Supplementary Figure S1. The prognostic correlation analysis of the m6A regulatory genes by Cox analysis method identified a correlation between *RBM15*, *IGFBP3*, *HNRNRPPC*, *HNRNPA2B1*, *IGFBP1*, *IGFBP2*, and *LRPPRC* and prognosis (Supplementary Table S2). Next, we conducted a survival analysis of the m6A regulatory genes. Each m6A regulator gene was assigned to high- or a low-expression group according to the optimal cutoff value in the stomach adenocarcinoma tissue. The results showed that the groups with low expression of *FTO*, *IGFBP3*, *IGFBP2*, *IGFBP1*, and *ZC3H13* had better survival rates than those patients in the high-expression group, while those with high expression of *RBMX*, *HNRNPA2B1, LRPPRC*, *FMR1*, *HNRNPC*, *YTHDF2*, *YTHDC2*, *RBM15B*, *RBM15*, *WTAP*, and *METTL3* had better survival rates than patients in the low-expression group (Supplementary Figure S2). The m6A prognostic network illustrated that the expression of most genes are positively correlated among writers, readers, and erasers, except that *YTHDF3*, *IGFBP2*, *LRPPRC*, and *IGFBP3* were negatively correlated among writers, readers, and erasers (Figure 2A). The non-negative matrix factorization (NMF) clustering has a consensus with respect to the expression of m6A regulators. Based on the consensus, matrix k-3 was an optimal clustering stability value; finally, three different clusters were identified (Supplementary Figure S3): m6Acluster-A (*n* = 317), m6Acluster-B (*n* = 204), and m6Acluster-C (*n* = 283). Figure 2B shows that m6Acluster-C has a better survival advantage, while m6Acluster-A has a poor prognosis (*p* = 0.005) among the three clusters.

### Distinct Immune Landscapes in m6A Modification Patterns

**FIGURE 3:**
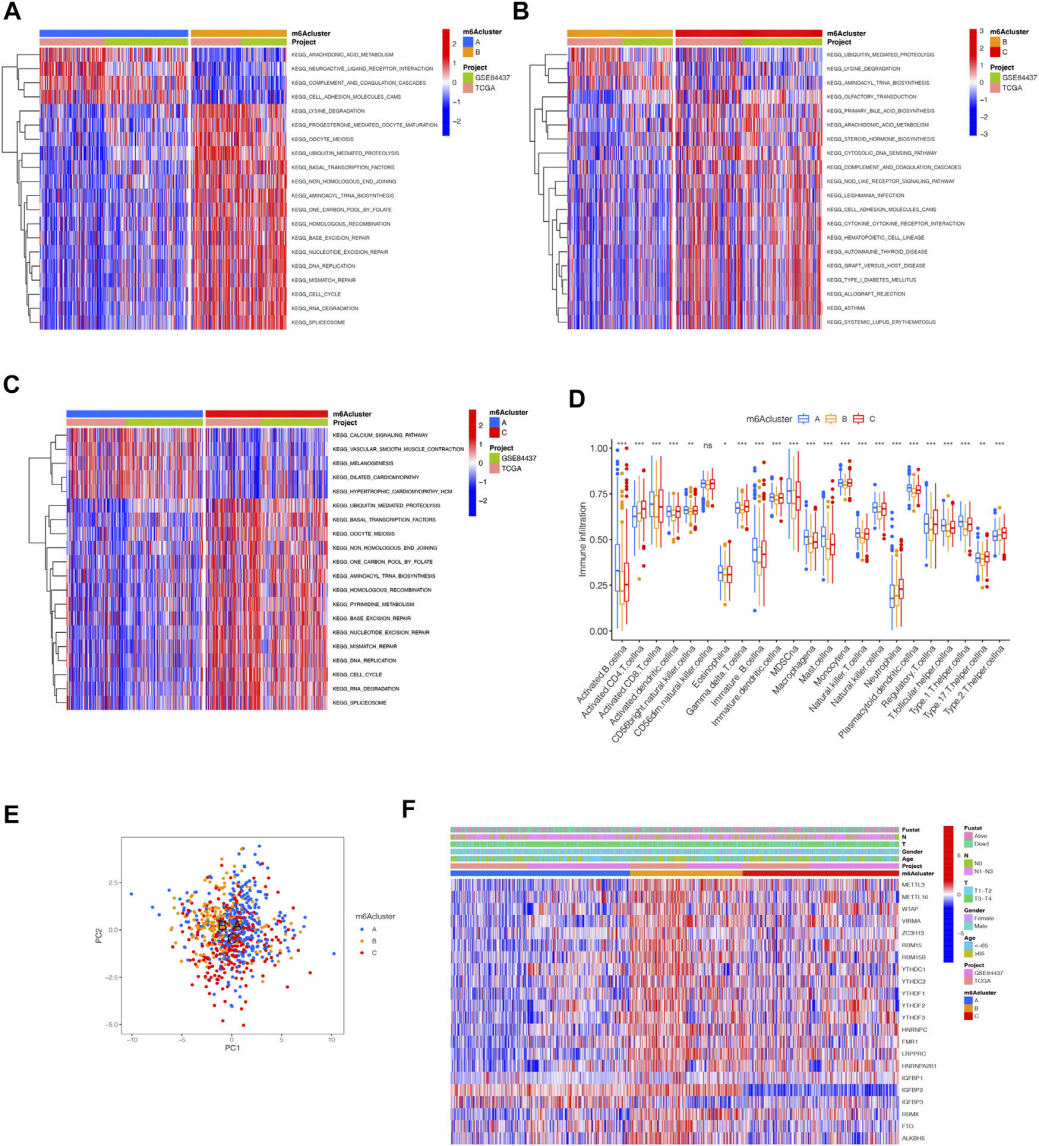
Distinct immune landscapes in m6A modification patterns and the biological characteristics of each pattern. **(A–C)** GSVA analyzed the differences between functional pathways in m6A modification patterns (adjusted *p*-value < 0.05). **(A)**, m6Acluster A vs. m6Acluster B; **(B)**, m6Acluster B vs. m6Acluster C; **(C)**, m6Acluster A vs. m6Acluster C. **(D)** Differential expression analysis of 23 immune cells among three m6A modification patterns. The asterisks represented the statistical *p* value (^∗∗∗^ *p* < 0.0001, ^∗∗^ *p* < 0.01, ^∗^ *p* < 0.05). **(E)** Scatter plot of PCA for m6A methylation modification pattern. **(F)** Unsupervised clustering of 23 m6A regulators of STAD.

By using GSVA, m6Acluster-A was significantly enriched in environmental information processing and signaling interaction; m6Acluster-B was significantly enriched in the immune system and biosynthesis of other secondary metabolites; m6Acluster-C was significantly enriched in environmental information processing and signal transduction (Figures 3A–C). Next, we used ssGSEA, which showed a rich innate immune cell immersion in m6Acluster-A. Figure 3D shows significant differences in the penetration characteristics of TME cells in the three clusters. The PCA results showed significant differences between the transcriptomes of the three m6A modification patterns (Figure 3E). The typed heat map of m6A revealed that the m6A-related genes were highly expressed in cluster-B and lowly in clusters A and C (Figure 3F).

### m6A-Related Genes’ Functional Annotation

**FIGURE 4:**
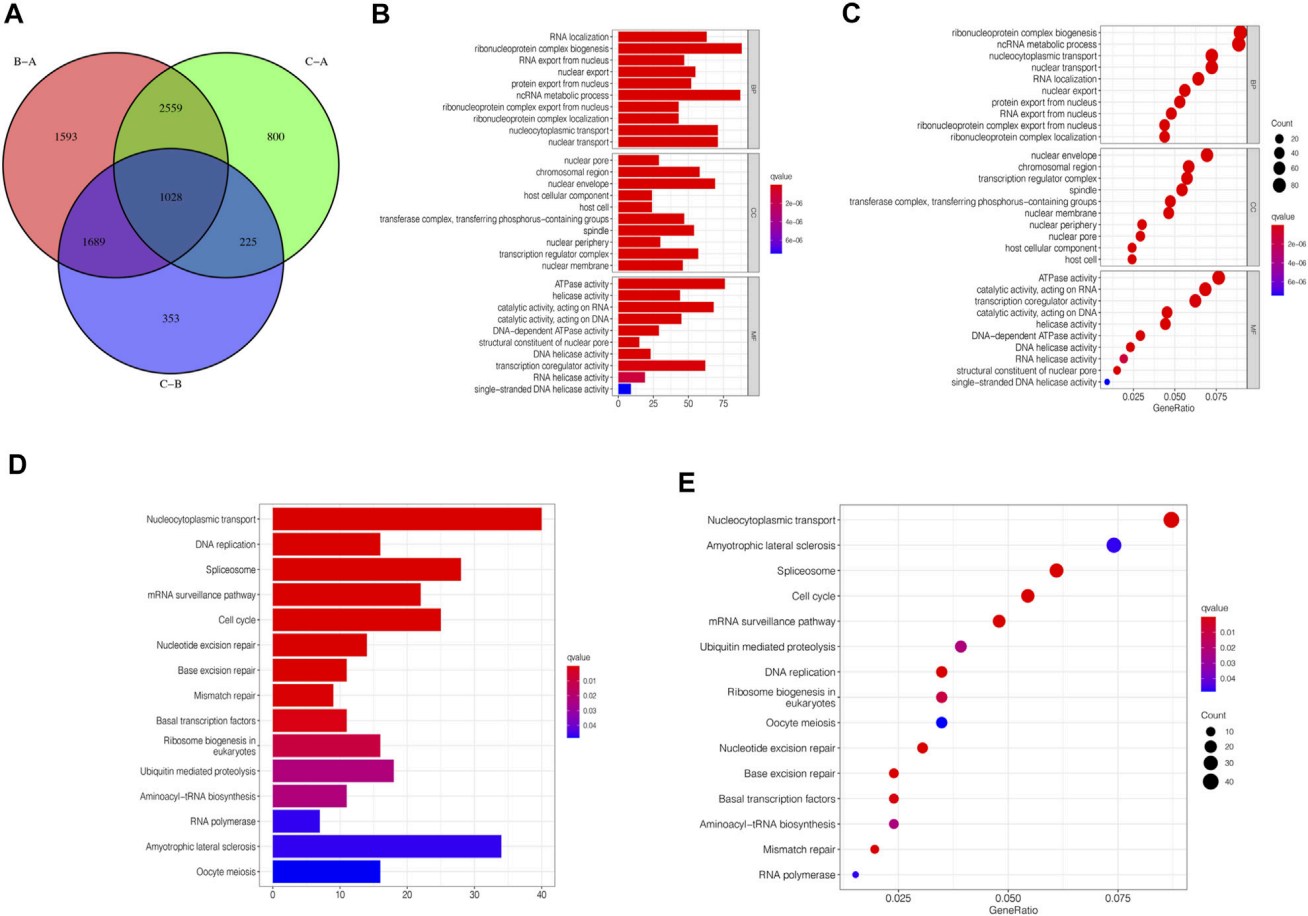
m6A-related genes’ functional annotation. **(A)** 1028 m6A-related DEGs between three m6A clusters are shown in the Venn diagram. **(B)** Functional annotation for m6A-related genes using GO enrichment analysis on the bar chart. **(C)** Functional annotation for m6A-related genes using GO enrichment analysis on the bubble chart. **(D)** Functional annotation for m6A-related genes using KEGG enrichment analysis on the bar chart. **(E)** Functional annotation for m6A-related genes using KEGG enrichment analysis on the bubble chart.

Although we divided STAD patients into three gene clusters based on the consensus clustering algorithm, the correlation between the m6A-related genes was not clarified. Hence, we analyzed the differences between m6A gene clusters (Figure 4A) and identified 1028 DEGs (Supplementary Table S2). The GO enrichment bar and bubble charts showed that the differential genes occurred in almost all cellular functions. In biological process (BP), the main enrichment was in RNA localization; in cellular component (CC), it was in the nuclear pore; in molecular function (MF), the main enrichment was in the ATPase activity (Figures 4B,C). The KEGG enrichment bar and bubble charts exhibited the involvement of the differential genes in the signaling pathway of nucleocytoplasmic transport (Figures 4D,E).

### Identification of m6A-Related Genes’ Phenotypes and m6A Scores

**FIGURE 5:**
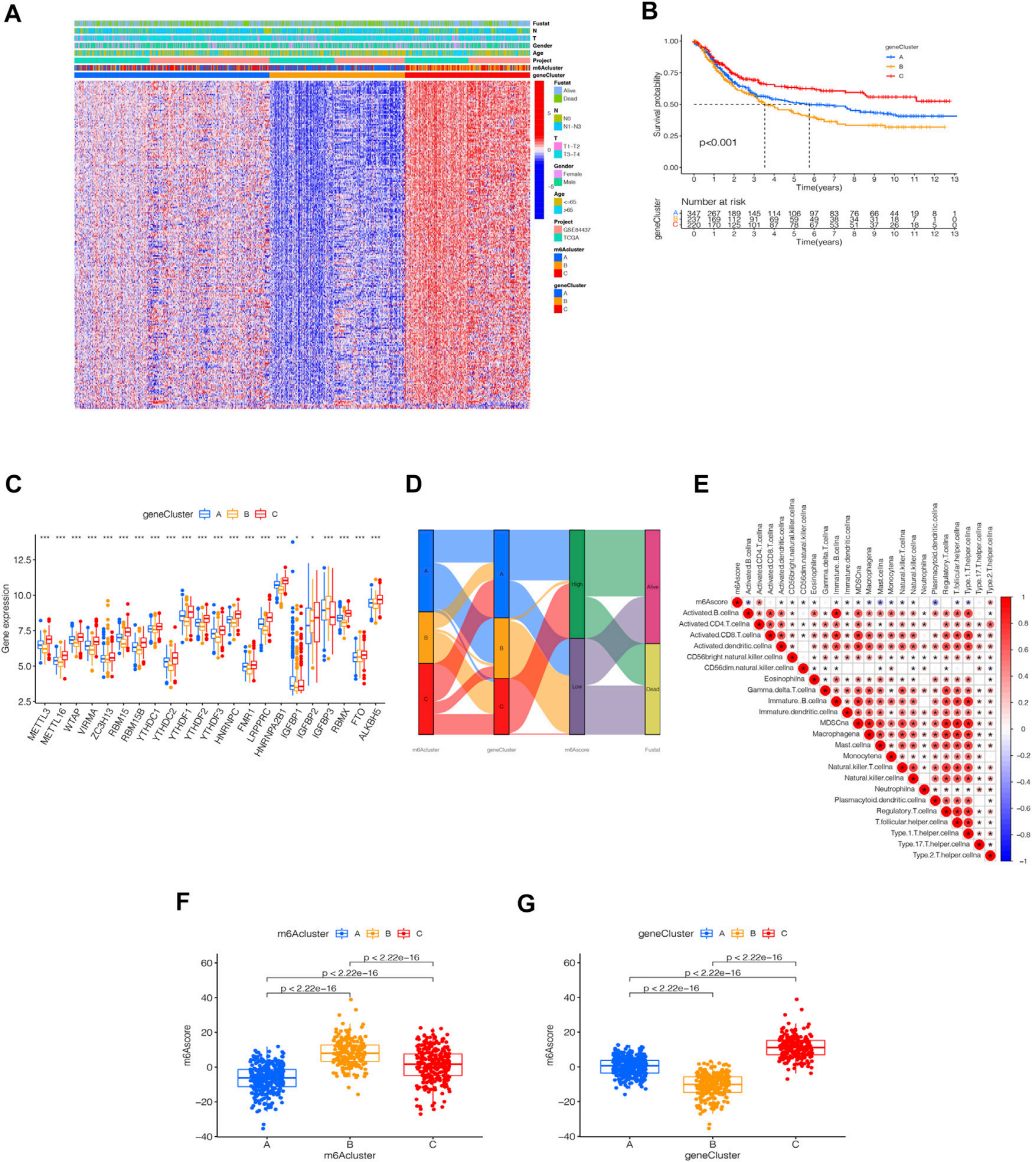
Identification of m6A-related genes’ phenotypes and m6A scores. **(A)** Heat map of genetic modification patterns. **(B)** Survival curves of different geneclusters (*p* < 0.0001, Log-rank test). **(C)** Box plot of the differential expression analysis of m6A-related genes among different geneclusters. The asterisks represented the statistical *p* value (^∗∗∗^ *p* < 0.0001, ^∗∗^ *p* < 0.01, ^∗^ *p* < 0.05). The one-way ANOVA test was used to test the statistical differences among three gene clusters. **(D)** Sankey diagrams of different genotypes. **(E)** Correlation analysis between the m6A score and immune cells, with red indicating positive correlation and blue indicating a negative correlation. The Kruskal-Wallis test was used to compare the statistical difference between three gene clusters (*p* < 0.001). **(F)** Differential expression analysis of the m6A score in the m6A cluster. **(G)** Difference analysis of m6A score in genecluster (*p* < 0.001, Kruskal-Wallis test)

To further analyze the DEGs associated with m6A phenotypes, we used a single-factor Cox method to identify the differential genes associated with STAD prognosis. Similar to the m6A modification pattern, we classified the m6A genomic phenotype into three categories: genecluster-A (*n* = 347), genecluster-B (*n* = 237), and genecluster-C (*n* = 220) (Supplementary Figure S4). The genecluster heat map containing clinical information showed a high expression of genecluster-C and low expression of genecluster-B (Figure 5A). The survival analysis of the three groups revealed that genecluster-B had the worst prognosis (*p* < 0.001) (Figure 5B). The difference analysis of genotype m6A showed significant differences among the three genotypes (Figure 5C). Considering the complexity of the quantification of m6A modification, we illustrated the workflow of m6A score construction with the Sankey diagram (Figure 5D). Next, we then rated m6A based on m6A correlation characteristics and divided it into the high m6Ascore group and the low m6Ascore group. The results of immunocyte difference analysis using m6A scores showed a significant positive correlation between m6A scores and activated CD4.T.cellna (Figure 5E). The results of the m6A score difference analysis showed that the scores were expressed in both the m6A cluster and the genecluster. The m6Acluster-B had the highest m6A score among the m6Aclusters (Figure 5F), while the genecluster-C had the highest score among geneclusters (Figure 5G).

### m6A Scores’ Clinical Prognosis Analysis and Somatic Tumor Mutations

**FIGURE 6:**
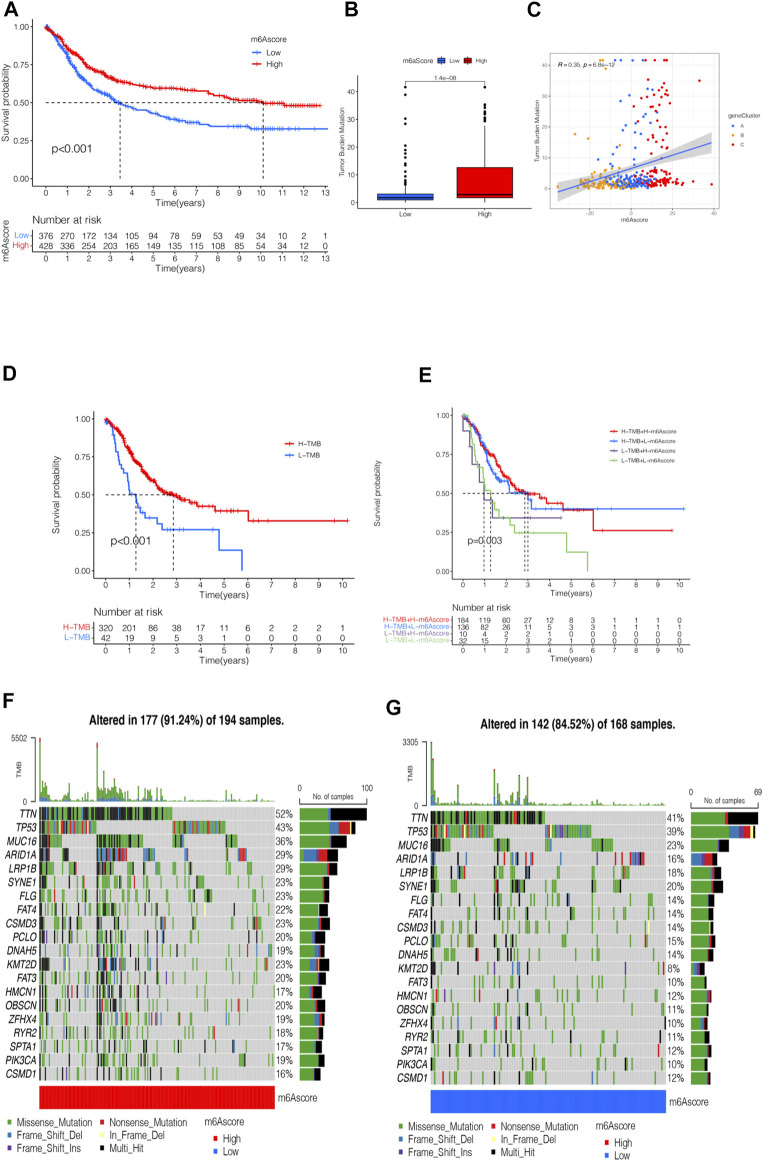
m6A scores’ clinical prognosis analysis and somatic tumor mutations. **(A)** Survival analysis of high- and low-m6A score groups using Kaplan-Meier curves (*p* < 0.001, Log-rank test). **(B)** Stratified analysis of the m6A score for STAD patients by tumor mutation burden (*p* < 0.001, Wilcoxon test). **(C)** A scatter plot describing the positive correlation between the m6A score and TMB. **(D)** Survival analysis of TMB (*p* < 0.001, Log-rank test). **(E)** Survival analysis of TMB combined with m6A score (*p* = 0.003, Log-rank test). **(F)** Waterfall chart of the high-m6A score group. **(G)** Waterfall chart of the low-m6A score group.

The survival analysis of the m6A score found that patients in the high m6A score group had a better prognosis than those in the low score group (Figure 6A). The analysis of the m6A score and TMB revealed a difference between patients in the high m6A score group and those in the low m6A score group; patients in the high m6A score group had high TMB (*p* < 0.001, Figure 6B). The correlation analysis showed a significant positive correlation between the m6A score and TMB (R = 0.35, *p* = 6.8e-12, Figure 6C). The survival analysis of TMB found that patients with a high number of mutations had a better survival duration than those with low mutations (*p* < 0.001, Figure 6D). Further analysis of the survival curve combining TMB and m6A scores found that patients had a significantly lower prognosis in the low tumor mutant and the low m6A score group (*p* = 0.003, Figure 6E). The STAD samples of the m6A score groups were analyzed based on the significant mutant gene (SMG). It was found that TTN and TP53 had high somatic mutation rates in both m6A score groups and high somatic mutation rates in the high m6A score groups (Figures 6F,G).

### Clinical Evaluation of m6A Scores

**FIGURE 7:**
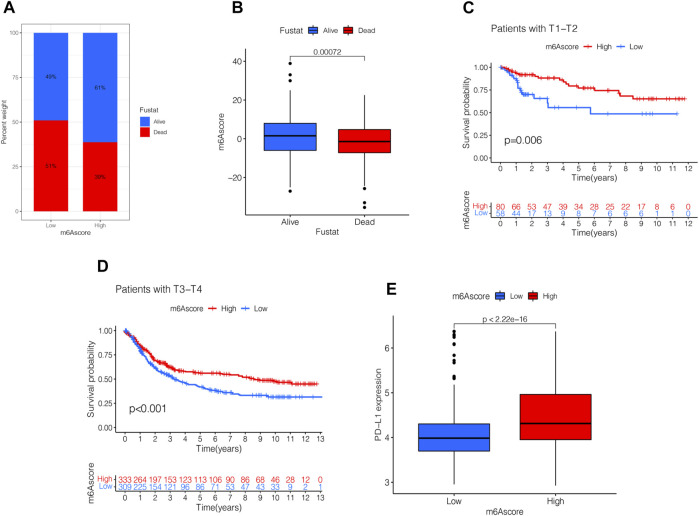
Validation and application of the m6A score in clinical evaluation. **(A)** Proportion of survival and death in high- and low-m6A score groups. **(B)** Comparison of the m6A score between survival and dead patients (*p* < 0.001, Wilcoxon test). **(C)** Stratified analysis of the m6A score for STAD patients by T1-T2 (*p* = 0.006, Log-rank test). **(D)** Stratified analysis of m6A score for STAD patients by T3-T4 (*p* < 0.001, Log-rank test). **(E)** Stratified analysis of m6A score for STAD patients by PD-L1 (*p* < 0.0001, Wilcoxon test).

Next, we analyzed the clinical relevance of the m6A score. As shown in Figure 7A, STAD patient deaths occurred in the low m6A score group. The rank test results showed that patients in the high m6A score group had prolonged survival (Figure 7B). To further analyze the clinical relevance, we divided the patients into the T1-T2 and the T3-T4 groups. The results of survival analyses of both groups showed that patients in the high m6A score group had a better prognosis than those in the low m6A score group (Figures 7C,D). To detect the differences in PD-L1 expression in the m6A score and support-related immunotherapy, we tested the expression of PD-L1 in the m6A score and observed that PD-L1 was significantly higher in the high m6A score group than in the low score group (*p* < 2.22e-16, Figure 7E).

### Role of the m6A Scores in Immunotherapy

**FIGURE 8:**
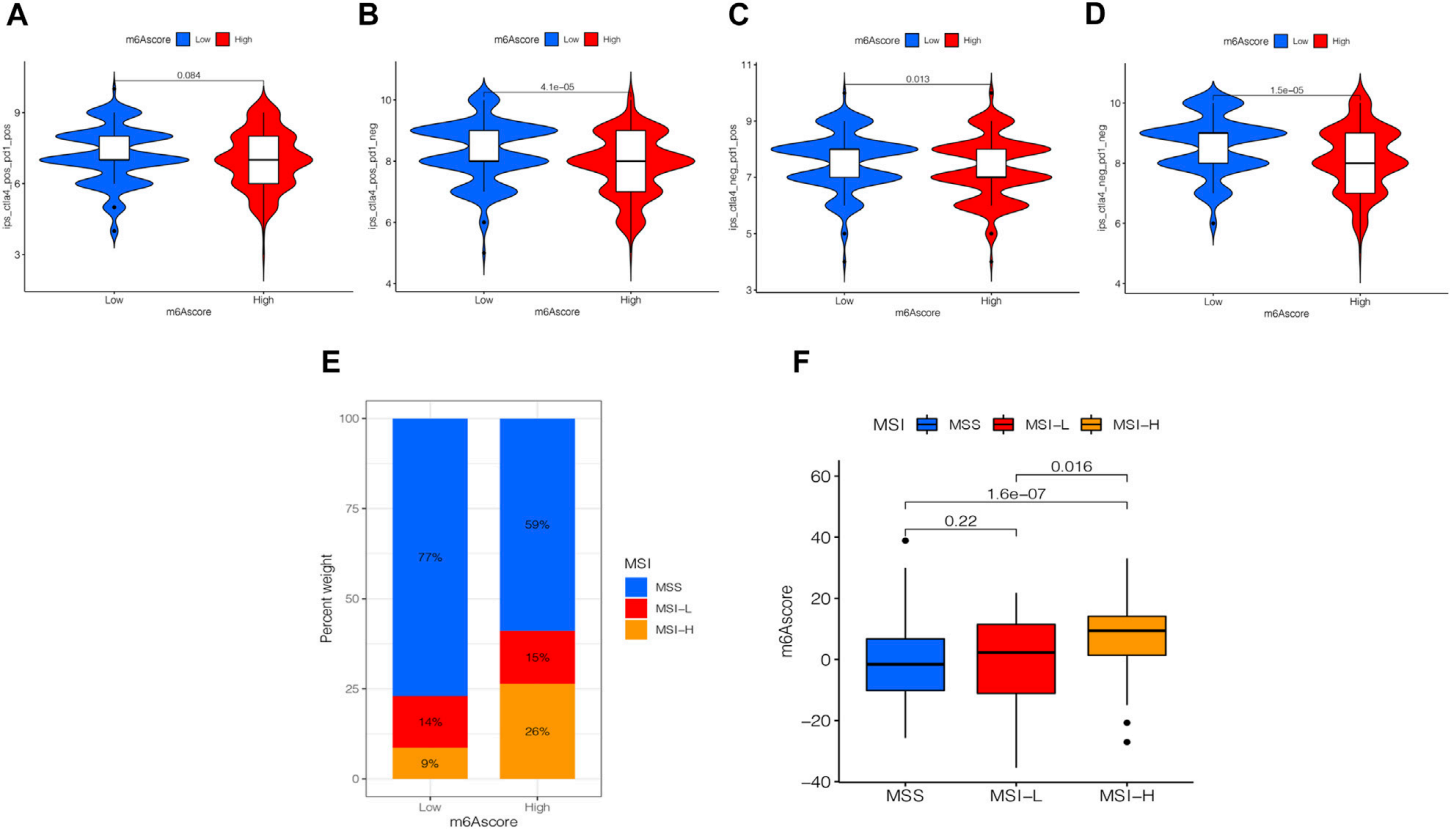
Analysis of the m6A score in anti-PD-L1 and CTLA-4 immunotherapy. **(A)** Differential analysis for the low m6A score group and the high m6A score group in immunophenoscore (IPS) with CTLA4 (+)/PD1 (+) (*p* = 0.084, Wilcoxon test). **(B)** Differential analysis for the low m6A score group and the high m6A score group in IPS with CTLA4 (+)/PD1 (−) (*p* < 0.0001, Wilcoxon test). **(C)** Differential analysis for the low m6A score group and the high m6A score group in IPS with CTLA4 (−)/PD1 (+) (*p* = 0.013, Wilcoxon test). **(D)** Differential analysis for the low m6A score group and the high m6A score group in IPS with CTLA4 (−)/PD1 (−) (*p* < 0.0001, Wilcoxon test). **(E)** The proportion of the m6A score in groups with high or low MSI and stable status. **(F)** Differences in the m6A score among high or low MSI and stable status. The differences between the three groups were compared through the Kruskal-Wallis test. MSS, microsatellite stable; MSI-H, high microsatellite instability; MSI-L, low microsatellite instability.

Presently, immunotherapy is becoming a prominent treatment method. Anti-HER-2 antibodies, anti-VEGF antibodies, tyrosine-kinase inhibitor (TKI), and immuno-checkpoint inhibitors (ICIs) have achieved preliminary results in the treatment of STAD. Thus, we tested the expression of IPS and MSI in the m6A score to predict the patient’s response to ICI treatment. Figure 8A shows that the IPS of the m6A score was not significantly different in CTLA-4/PD-1 immunotherapy in two groups. In the other three groups, the IPS of the low m6A score group increased significantly compared to the high m6A score group (Figures 8B–D). To explore the critical clinical significance of chemotherapy response to MSI gastric cancer, we divided MSI into three groups, high-frequency microsatellite instability (MSI-H), low-frequency microsatellite instability (MSI-L), and microsatellite stability (MSS), according to the MSI diagnostic criteria proposed by the Cancer Institute (NCI). As seen in Figure 8E, MSS was high in the high and low m6A score groups. The MSI-H subtype score was significantly different from the other two groups and had a higher score (Figure 8F).

## Discussion

m6A modification plays an important role in gene regulation and tumor development (Zhao et al., 2017). Overexpression or low expression of m6A-related genes can alter m6A modification in tumors and affect tumor development (Chen et al., 2019; Shulman and Stern-Ginossar, 2020). Thus, understanding the molecular mechanism of m6A modification and identifying the abnormal expression of m6A regulatory factors in clinical biopsy specimens is crucial for the clinical treatment and prognosis of early tumor diagnosis. Although the function of m6A in different cell types and microenvironments is being revealed gradually, the role of multiple m6A regulators in TME cell infiltration and the molecular mechanism of the anti-tumor immune response is yet unclear. Therefore, STAD immunotherapy was explored with respect to the characteristics of TME cell infiltration in different m6A modification patterns.

In the current study, we used 23 m6A methylation-related genes and found three types of m6A methylation modification patterns that differed significantly in immune infiltration. m6Acluster-A had high immune cell number and lymphocyte infiltration, m6Acluster-B was involved in the Wnt, TGF-β, JAK2, and other signaling pathways, and m6Acluster-C was deficient in immune cell infiltration. These three types of m6A methylation modification patterns correspond to immune-inflammatory type (immune inflamed), immuno-exclusion type (immune exclusive), and immune desert type (immune desert), respectively (Chen and Mellman, 2017). A comprehensive analysis of the infiltration characteristics of TME cells in the m6A methylation modification pattern in STAD provided a new strategy for exploring STAD-targeted therapeutic drugs. The immune inflammatory tumors are referred to as tumors with high levels of PD-L1 expression in cancer cells and excess immune cells and tumor-insulated lymphocytes (TILs) in the tumor. In the trials of PD-1/L1 inhibitor monotherapy of non-small cell lung cancer, such as KEYNOTE-042 and IMpower110, patients with high PD-L1 expression (≥50%) were likely to have prolonged survival (Mok et al., 2019; Herbst et al., 2020). Even in the high expression subgroup, the objective remission rate (ORR) of treatment was only about 40%. Therefore, the inflammatory tumors need to be investigated in-depth to improve the benefits of treatment. For example, previous studies suggested that PD-L1 had a high expression, TILs were fully infiltrated, and immunotherapy was effective. However, subsequent studies hinted that it might be necessary to distinguish between highly expressed PD-L1 in TME and immune or cancer cells. If PD-L1 came from immune cells, there were more TILS in the TME, especially CD8-T cells. On the other hand, TILs were abundant in the tumor, tumor and matrix interface, and matrix. Thus, optimal response conditions were created for immunotherapy, such that patients may have a better response to the treatment of PD-1/L1 inhibitors. Conversely, if PD-L1 came from tumor cells, TILs were mostly immersed in the matrix around the tumor. Therefore, the infiltration characteristics of TME cells in m6A methylation modification patterns provided potential therapeutic targets and novel ideas for the prevention of STAD.

In addition, we identified DEGs based on the m6A methylation modification patterns. Further analysis showed that these DEGs had m6A-related characteristic genes closely related to tumor prognosis and immune pathways. These m6A-related characteristic genes were genotyped according to the cluster analysis. The results of the analysis of the phenotype genes showed that they were closely related to cell-matrix and immune activation, which further validated the role of m6A methylation in TME. Then, we adjusted the mRNA levels according to m6A regulatory genes and classified them into high- and low-expression groups according to the mRNA expression median value. Subsequently, the m6A score model was constructed to evaluate the m6A modification patterns in individual patients with STAD, and the effects of individual heterogeneity were excluded. This provided accurate guidance for immunotherapy in patients with STAD. According to the m6A score difference analysis results, m6Acluster-B with immuno-exclusion type had the highest m6A score, while m6Acluster-A with immune-inflammatory type had the lowest m6A score. The immuno-correlated analysis established a positive correlation between m6A scores and CD4^+^ T cells. These results showed that the m6A score could determine the TME-infiltrated tumor immunophenotype and guide precision immunotherapy in patients with STAD. Several studies have reported that the higher the TMB in cancer patients, the better the prognosis (Cheng et al., 2015; Li et al., 2016; Kelly, 2017; Wei et al., 2021). Therefore, TMB can be used as a predictive biomarker for patients with STAD during ICI progression, facilitating clinical decision-making. Our analysis found that the m6A score was significantly positively correlated with TMB, which was consistent with previous findings. Subsequent studies found a correlation between the m6A score in mutation burden, PD-L1 expression, and MSI state. Additionally, the predictive advantages of the m6A score in immunotherapy of patients with STAD were determined.

A large number of studies have found that m6A-related genes play a major role in the progression and metastasis of STAD. METTL3 was the main catalytic component of methyl transfer enzyme complexes, and its abnormal expression can alter the expression of *m6A* mRNA, affecting the proliferation, metastasis, invasion, and apoptosis of STAD. Yue et al. (2019) demonstrated that elevated METTL3 expression was positively correlated with poor prognosis in patients and thus contributed to the epithelial-mesenchymal transition process and metastasis. Jiang et al. (2020) demonstrated that METTL3 knockout increases the expression of suppressor of cytokine signaling (SOCS) protein families in STAD cells and that SOCS2 expression is negatively correlated with STAD cell proliferation. This phenomenon suggested that a decline in METTL3 elevates SOCS2 expression and inhibits STAD cell proliferation. A recent study found that m6A and METTL3 expression levels increased in STAD and that elevated METTL3 expression indicated high malignancy and poor prognosis in patients (Sun et al., 2020). FTO was the first m6A methylation enzyme to be discovered. Some studies found that FTO was associated with STAD development and might be a vital molecular target for monitoring STAD prognosis. Zhang et al. (2019) demonstrated that low m6A signals were associated with poor clinicopathological characteristics of STAD. The mechanism studies revealed that FTO overexpression could reduce m6A methylation levels, activate Wnt/PI3K-AKT pathways, and promote malignant phenotypes of STAD. The YTH family protein is bound to mRNA containing m6A, which regulates the positioning and stability of mRNA. This family of proteins was associated with the development of STAD. Based on the biological information from various human cancer databases, one study found that about 7% of STAD patients had YTHDF1 mutations and that high expression of YTHDF1 was associated with high tumor proliferation rates and poor overall survival (Pi et al., 2021). *In vivo* and *in vitro* experiments confirmed that YTHDF1 promotes the translation of the Wnt pathway key receptor protein frizzled 7 (FZD7) in a m6A-dependent manner and enhanced the expression of FZD7. Subsequently, the Wnt/β-catenin signaling pathway was triggered; which facilitated the occurrence of STAD. These results confirmed our findings and demonstrated that m6A-related genes in TME play a critical role in the metabolism, drug resistance, and metastasis of STAD, suggesting that m6A modification can be used as a target for the prevention and treatment of STAD.

The systematic study on the m6A score revealed its role in gastric cancer patients in clinical practice. First, the m6A score could be used to evaluate m6A methylation patterns in patients with STAD, which elaborated the corresponding TME cell infiltration characteristics. This enhanced our understanding of the immune phenotype of STAD, thereby improving the clinical treatment conversion effect. In this study, we found that the m6A score was closely related to the clinicopathological characteristics of STAD, including TNM, TMB, and MSI. Therefore, the m6A score can be used as an independent prognostic biomarker for STAD to guide clinical treatment, as well as a supplementary assessment criterion for immunotherapy to predict the clinical effects of immunotherapy. Furthermore, it can also be used as a sensitive index of precision immunotherapy for STAD. Importantly, the present study confirmed the role of m6A regulatory factors or m6A phenotype-related genes in STAD. Targeting these genes can alter the characteristics of TME cell infiltration and improve the effectiveness of targeted immunotherapy, thereby opening a new avenue for epigenetics and tumor research and the underlying regulatory mechanisms.

## Conclusion

In summary, this study systematically evaluated the modification patterns of 23 m6A regulatory factors in STAD. Thus, it was proved that different modification patterns may be critical factors leading to inhibitory changes and heterogeneity in TME. This will elucidate TME infiltration characteristics in patients with STAD based on the evaluation of m6A modification patterns. This promoted basic research in relevant areas and created opportunities for clinical STAD patients to predict prognosis and explore novel immunotherapies.

## Data Availability

The datasets presented in this study can be found in online repositories. The names of the repository/repositories and accession number(s) can be found in the article/Supplementary Material.
