# Supplementary material for: N6-Methyladenosine Modification Patterns and Tumor Microenvironment Immune Characteristics Associated With Clinical Prognosis Analysis in Stomach Adenocarcinoma
Source: Front Cell Dev Biol. 2022 Jun 15;10:913307. doi: 10.3389/fcell.2022.913307 (PMC9261346; doi:10.3389/fcell.2022.913307)
Supplement: Supplementary file 1 [file Table1.DOCX]

**Table S1.** The genes in the top 10 of the tumor mutant burden

| **Gene** | **Number** |
| --- | --- |
| TTN | 208 |
| TP53 | 176 |
| MUC16 | 133 |
| ARID1A | 103 |
| LRP1B | 102 |
| SYNE1 | 93 |
| FLG | 84 |
| FAT4 | 82 |
| CSMD3 | 79 |
| PCLO | 72 |
