# Supplementary figures and images for: N6-Methyladenosine Modification Patterns and Tumor Microenvironment Immune Characteristics Associated With Clinical Prognosis Analysis in Stomach Adenocarcinoma
Source: Front Cell Dev Biol. 2022 Jun 15;10:913307. doi: 10.3389/fcell.2022.913307 (PMC9261346; doi:10.3389/fcell.2022.913307)

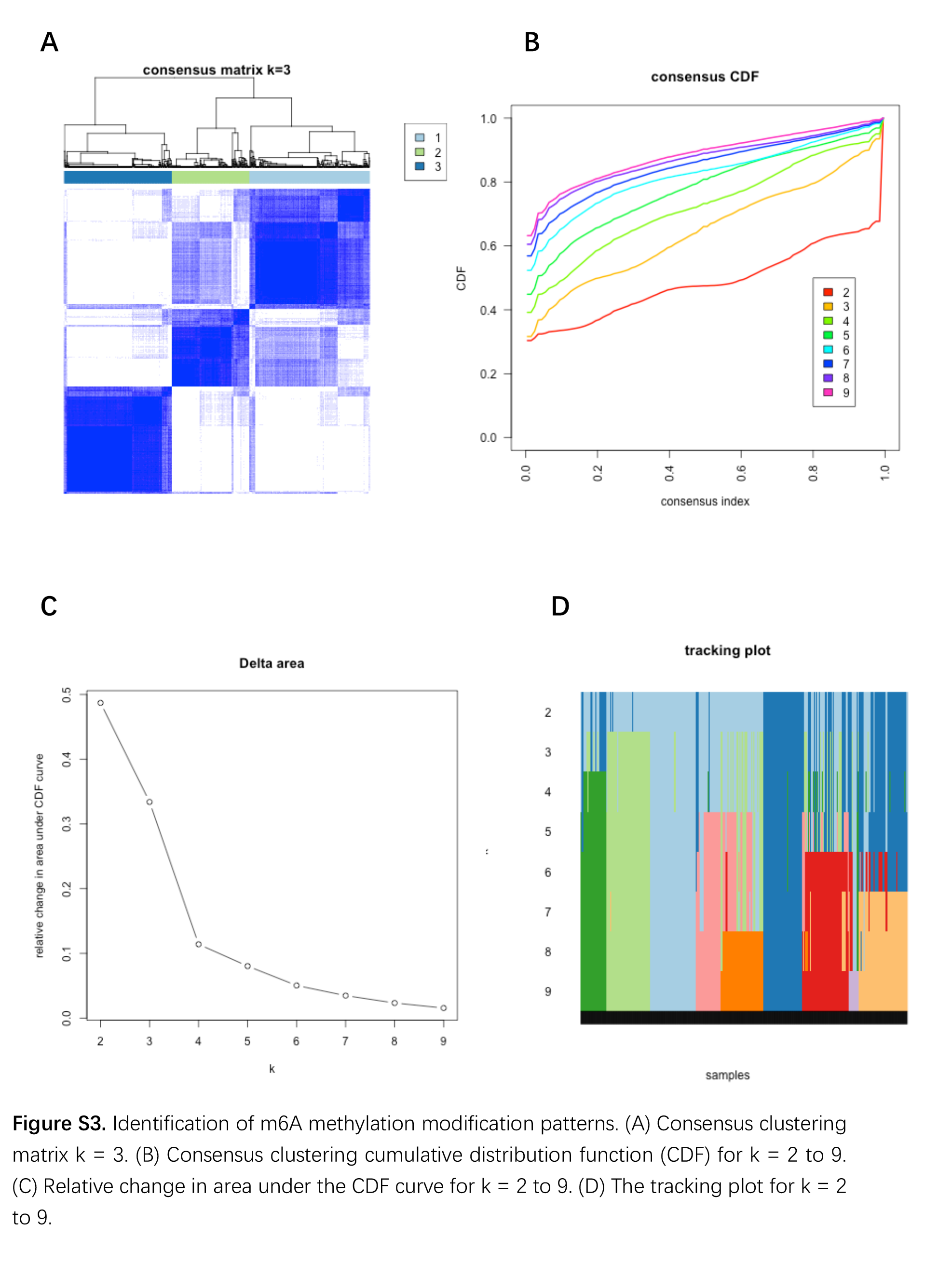

Supplement: Supplementary file 2 [file Image3.TIF]

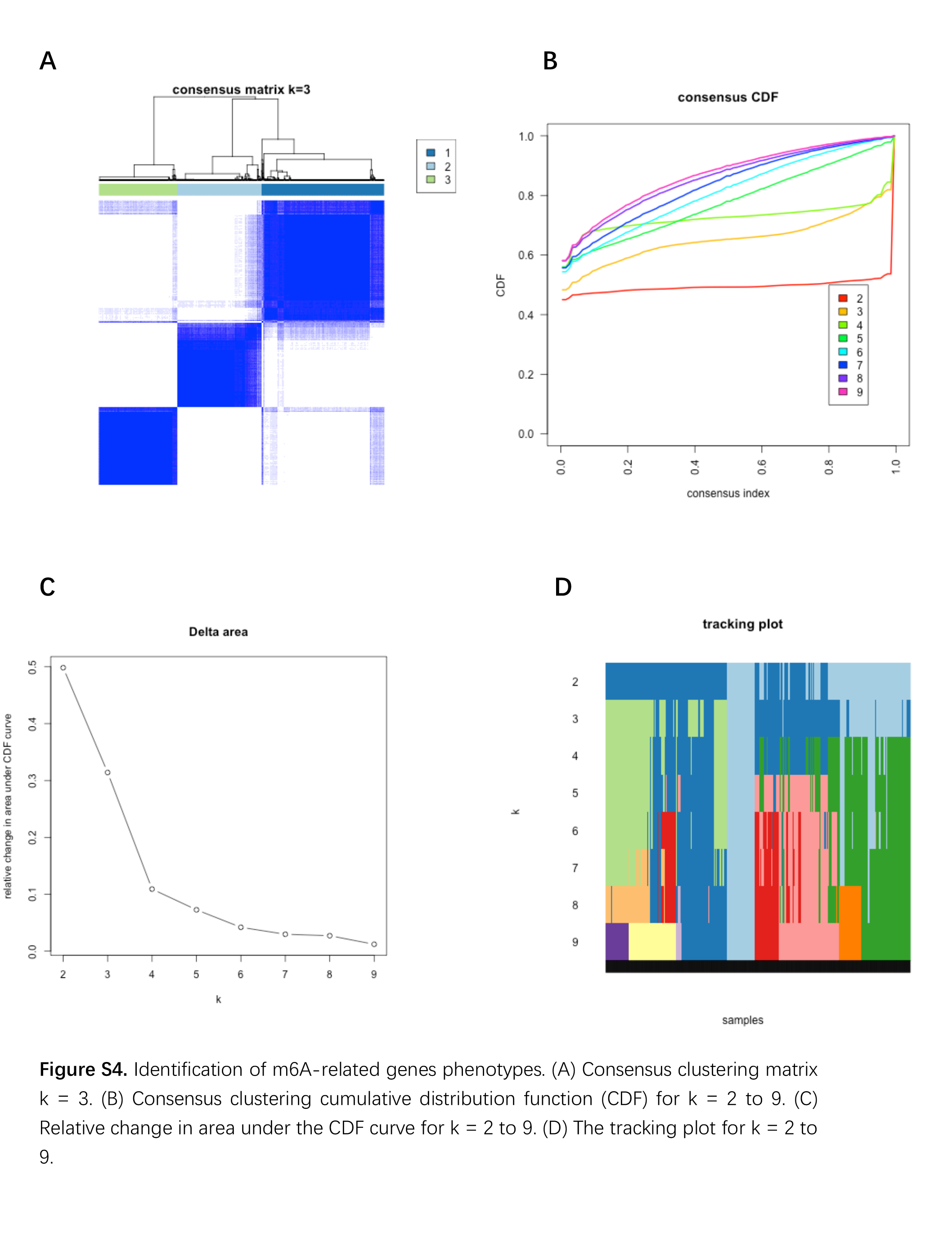

Supplement: Supplementary file 3 [file Image4.TIF]

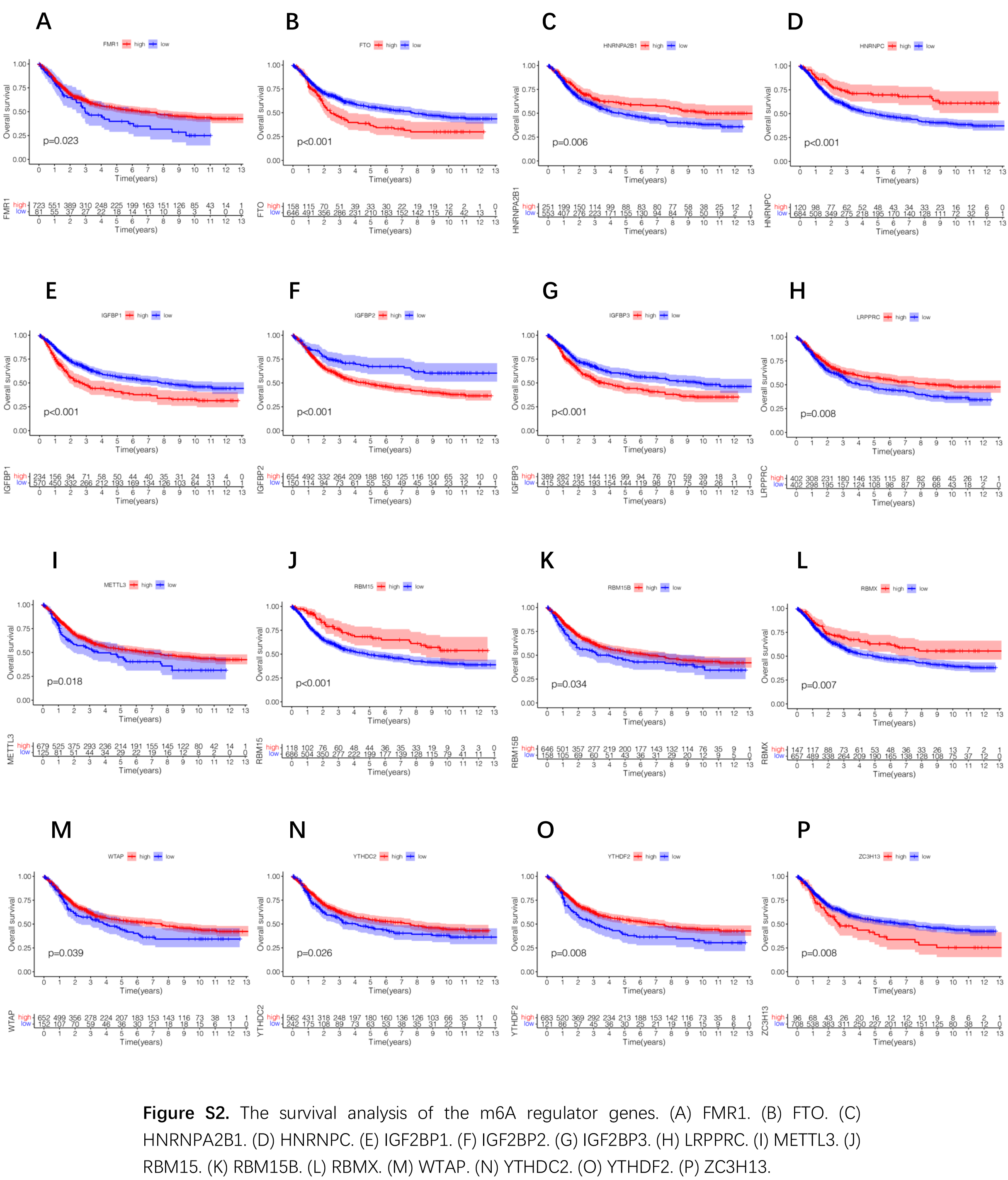

Supplement: Supplementary file 5 [file Image2.TIF]

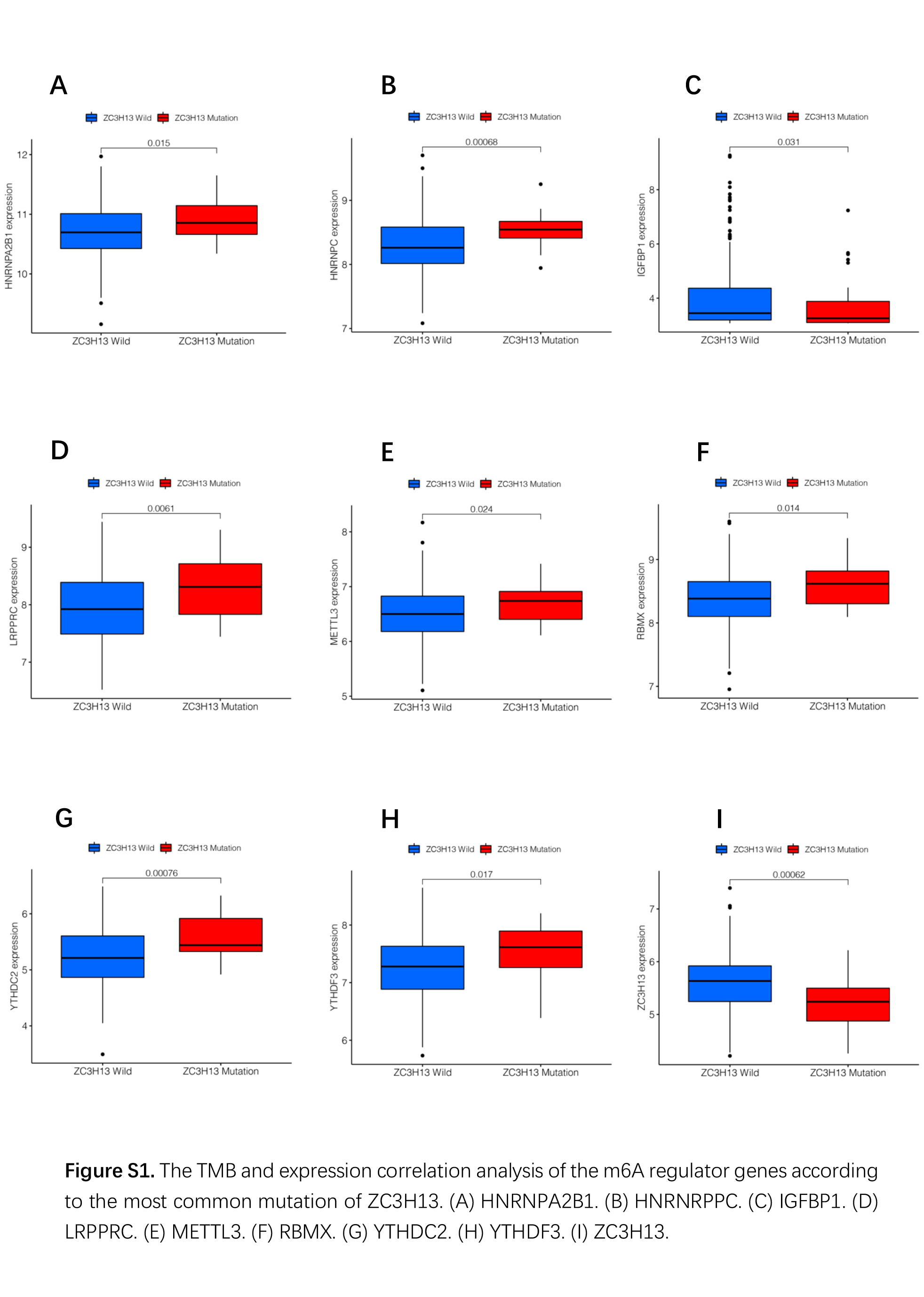

Supplement: Supplementary file 6 [file Image1.TIF]
