# Supplementary material for: N6-Methyladenosine Modification Patterns and Tumor Microenvironment Immune Characteristics Associated With Clinical Prognosis Analysis in Stomach Adenocarcinoma
Source: Front Cell Dev Biol. 2022 Jun 15;10:913307. doi: 10.3389/fcell.2022.913307 (PMC9261346; doi:10.3389/fcell.2022.913307)
Supplement: Supplementary file 7 [file Table2.DOCX]

**Table S2.** Univariate Cox regression analysis of differential genes in STAD

| **GENE** | **HR** | **95%-L** | **95%-H** | **p value** |
| --- | --- | --- | --- | --- |
| RBM15 | 0.6933 | 0.5638 | 0.8525 | 0.0005 |
| IGFBP3 | 1.1702 | 1.0658 | 1.2848 | 0.0010 |
| HNRNPC | 0.6752 | 0.5333 | 0.8549 | 0.0011 |
| HNRNPA2B1 | 0.7318 | 0.5764 | 0.9292 | 0.0104 |
| IGFBP1 | 1.0980 | 1.0194 | 1.1828 | 0.0137 |
| IGFBP2 | 1.0787 | 1.0121 | 1.1497 | 0.0198 |
| LRPPRC | 0.8386 | 0.7041 | 0.9987 | 0.0483 |
| RBMX | 0.7878 | 0.6153 | 1.0086 | 0.0585 |
| FTO | 1.1849 | 0.9773 | 1.4366 | 0.0844 |
| YTHDC2 | 0.8380 | 0.6849 | 1.0254 | 0.0861 |
| YTHDF2 | 0.8385 | 0.6665 | 1.0548 | 0.1325 |
| WTAP | 0.8295 | 0.6425 | 1.0709 | 0.1515 |
| ZC3H13 | 1.1318 | 0.9264 | 1.3828 | 0.2256 |
| METTL3 | 0.8808 | 0.7051 | 1.1003 | 0.2636 |
| RBM15B | 0.8969 | 0.7348 | 1.0946 | 0.2842 |
| FMR1 | 0.9104 | 0.7198 | 1.1515 | 0.4336 |
| METTL16 | 0.9276 | 0.7381 | 1.1657 | 0.5191 |
| YTHDC1 | 0.9306 | 0.7046 | 1.2292 | 0.6126 |
| YTHDF1 | 0.9686 | 0.8064 | 1.1635 | 0.7333 |
| YTHDF3 | 0.9685 | 0.7958 | 1.1787 | 0.7494 |
| VIRMA | 0.9918 | 0.8022 | 1.2264 | 0.9397 |
| ALKBH5 | 0.9926 | 0.7898 | 1.2473 | 0.9489 |

Abbreviation: L, low; H, high.
